# Cost-effectiveness analysis of direct admission to acute geriatric unit versus admission after an emergency department visit for elderly patients

**DOI:** 10.1186/s12877-023-03985-0

**Published:** 2023-05-10

**Authors:** Diane Naouri, Youri Yordanov, Nathanael Lapidus, Nathalie Pelletier-Fleury

**Affiliations:** 1 Centre for Research in Epidemiology and Population Health, French National Institute of Health and Medical Research (INSERM U1018), Université Paris- Saclay, Université Paris-Sud, UVSQ, Villejuif, France; 2 Service d’Accueil des Urgences, Sorbonne Université, APHP, Hôpital Saint Antoine, INSERM, Institut Pierre Louis d’Epidémiologie et de Santé Publique, UMR-S 1136, Paris, France; 3 Public Health Department, Saint-Antoine Hospital, Sorbonne Université, INSERM, Institut Pierre Louis d’Epidémiologie et de Santé Publique IPLESP, AP-HP, Paris, F75012 France

**Keywords:** Geriatrics, Hospital, Costs, Emergency department

## Abstract

**Background:**

Elderly individuals represent an increasing proportion of emergency department (ED) users. In the Greater Paris University Hospitals (APHP) direct-admission study, direct admission (DA) to an acute geriatric unit (AGU) was associated with a shorter hospital length of stay (LOS), lower post-acute care transfers, and lower risk of an ED return visit in the month following the AGU hospitalization compared with admission after an ED visit. Until now, no economic evaluation of DA has been available.

**Methods:**

We aimed to evaluate the cost-effectiveness of DA to an AGU versus admission after an ED visit in elderly patients. This was conducted alongside the APHP direct-admission study which used electronic medical records and administrative claims data from the Greater Paris University Hospitals (APHP) Health Data Warehouse and involved 19 different AGUs. We included all patients ≥ 75 years old who were admitted to an AGU for more than 24 h between January 1, 2013 and December 31, 2018. The effectiveness criterion was the occurrence of ED return visit in the month following AGU hospitalization. We compared the costs of an AGU stay in the DA versus the ED visit group. The perspective was that of the payer. To characterise and summarize uncertainty, we used a non-parametric bootstrap resampling and constructed cost-effectiveness accessibility curves.

**Results:**

At baseline, mean costs per patient were €5113 and €5131 in the DA and ED visit groups, respectively. ED return visit rates were 3.3% (n = 81) in the DA group and 3.9% (n = 160) in the ED group (p = 0.21). After bootstrap, the incremental cost-effectiveness ratio was €-4249 (95%CI= -66,001; +45,547) per ED return visit averted. Acceptability curves showed that DA could be considered a cost-effective intervention at a threshold of €-2405 per ED return visit avoided.

**Conclusion:**

The results of this cost-effectiveness analysis of DA to an AGU versus admission after an ED visit for elderly patients argues in favor of DA, which could help provide support for public decision making.

## Introduction

In many industrialised countries, access block as well as emergency department (ED) overcrowding, are well documented [1, 2]. We know that they are a source of additional morbi-mortality [3–5] and medical errors [6].

Elderly individuals represent an increasing proportion of those requiring admission to the ED and accounted for more than 2.7 million ED visits in 2019 in France [7, 8]. Advanced age brings a higher likelihood of presenting multiple chronic conditions, [9] and frailty [9, 10]. These conditions expose individuals to an increased risk of negative health-related outcomes such as disability, hospitalizations, institutionalization, and death [9]. Elderly patients often experience long waiting times in the ED [11, 12] and subsequent problems obtaining a hospital bed [13, 14]. This is particularly true for those living in institutions for whom an ED visit is identified as a possible source of aggravation [15, 16]. In a report published in 2018 in France, it was established that 45% of hospitalizations of the elderly were preceded by an ED visit [16].

One solution might be to avoid referring elderly patients to the ED and to promote direct admissions (DAs) to an acute geriatric unit (AGU) for those requiring hospitalization. Few studies have compared DAs to an AGU [17–19] with admissions after an ED visit. However, one study showed that admissions after an ED visit were more frequent in elderly patients with a previous history of arrythmia or protein-energy malnutrition, and were associated with a higher likelihood of post-acute care transfer [18]. In another study conducted among people living in nursing homes, admissions after being seen in an ED were more frequent among the most elderly [17]. In the Greater Paris University Hospitals (APHP) direct-admission survey [19], a multicenter retrospective cohort study using data from the APHP Health Data Warehouse between 2013 and 2018, the aim was to evaluate the benefits on morbidity of DA to an AGU compared with admission after an ED visit, for patients older than 75 years. The study showed that DA was associated with a shorter hospital length of stay (LOS) and that there were no significant associations with the risk of an ED return visit in the month following the AGU hospitalization. However, until now there has been no available economic evaluation of DA. Using data from the APHP direct-admission study, we aimed to evaluate the cost-effectiveness of DA to an AGU versus admission after an ED visit in elderly patients.

## Materials and methods

### Study design and setting

This economic evaluation was conducted alongside the APHP direct-admission study [19]. Briefly, APHP direct-admission was a retrospective cohort study which used the electronic medical records and administrative claims data from the APHP Health Data Warehouse [20]. It involved sizable data from 19 APHP AGUs covering, for example, demographics, standardised hospitalization reports (notably with information about living conditions and helpers), coded diagnoses (according to ICD-10), and therapeutic interventions (according to the French Common Classification of Medical Acts [CCAM]). Details regarding the APHP direct-admission study, as well as available data variables, have been described previously [19].

### Study participants [19]

All patients ≥ 75 years old admitted to an AGU for more than 24 h (inpatient care), between January 1st, 2013 and December 31st, 2018, were included in the APHP direct-admission study. When patients had been admitted several times, we analyzed their latest admission. We excluded all patients who were admitted to the AGU more than 5 days after an ED admission and those who were admitted after hospitalization to an intensive care unit and/or non-geriatric specialty unit. We also excluded all patients presenting at ED with clinical signs of life-threatening conditions (such as mottling, respiratory distress, cyanosis, indrawing, and need for vascular filling) and those with diagnoses that did not adhere to the positivity assumption of propensity score.

### Intervention

The intervention was DA to an AGU (DA group) as opposed to an admission after an ED visit (ED group), which was chosen as the reference strategy.

### Statistical analysis

Using propensity score modeling for DA and inverse-probability treatment weighting (IPTW, see below), patients directly admitted to the AGU were compared with patients admitted to the AGU after an ED visit.

#### Control of confounding

We performed multiple imputations in order to handle missing data [21], and the IPTW approach was used to balance the differences in baseline variables between intervention groups [19, 22]. Details regarding how multiples imputation, propensity score modeling, and balance diagnostics before and after imputation and inverse probability treatment weighting (IPTW), are available in the original paper of the APHP direct-admission study [19].

#### Health-economic evaluation

*Effectiveness criteria.* In this cost-effectiveness study, the effectiveness criterion was the occurrence of ED return visit in the month following AGU hospitalization.

*Cost analysis*. The cost analysis was conducted from the payer’s perspective, i.e., the National Health Insurance Fund (*Caisse Nationale d’Assurance Maladie*, [CNAM]). The time horizon was the time of the hospitalization. Related costs were direct medical costs charged by the hospital for the hospitalization in acute care (corresponding to AGU hospitalization as well as ED visit). The monetary valuation was made in euros at 2019 rates. For each patient, the duration (in days) of hospitalization was collected and valued. For valuations, data from the Program for the Medicalization of Information Systems (PMSI) was used through diagnosis-related groups (*Groupe Homogène de Malades* [GHM]) and their linked tariffs and stay-related groups (*Groupe Homogène de Séjours* [GHS]). In France, every type of stay is assigned to a GHM/GHS entity based on the principal diagnosis, procedures performed, LOS, and level of severity (comorbidities and complications). Costs per patient were expressed as median costs (1st and 3rd quartiles) per group. Given the length of follow-up, costs and outcomes were not discounted.

*Cost-effectiveness analysis.* These mean costs were combined with the rate of ED return visit in the month following AGU hospitalization to calculate incremental cost-effectiveness ratios (ICERs). ICERs reflect the additional cost needed to avoid one ED return visit, i.e., the cost per ED return visit averted. Statistical uncertainty surrounding the ICER was expressed with a 95% confidence interval estimated by 5000 non-parametric bootstrap replications. Variability of the ICER was illustrated by plotting a cost-effectiveness plane, where the reference was placed at the origin: the results appear as a scatter of 5000 possible outcomes, with each point representing a bootstrap replication. Results were interpreted with respect to the socially acceptable financial effort, i.e., in the case of our study, the threshold value the National Health Insurance Fund would be willing to pay for an additional unit of effectiveness. To facilitate the decision-making process, we plotted a cost-effectiveness acceptability curve (CEAC): the probability that a treatment is economically acceptable, given a specific cost-effectiveness threshold (i.e., the payer’s willingness to pay), is plotted on the y-axis over a wide range of possible thresholds of costs along the x-axis [23].

R software for Spark (SparkR) was used for analyses. The reporting of this study followed the Consolidated Health Economic Evaluation Reporting Standards (CHEERS) guidelines [24].

## Results

Among the 20,416 patients admitted to an AGU during our study period, 6583 were included in the study: 37.5% (n = 2470) in the DA group and 62.5% (n = 4113) in the ED group. The detailed flowchart with the original results is available in the original paper of the APHP direct-admission study [19]. There was no statistical difference between the two groups according to age, gender, comorbidities, functionality [19].

From all patients, ED return visit rates were 3.3% (n = 81) in the DA group and 3.9% (n = 160) in the ED group. When considering the cost of acute hospital stays in both groups, mean costs per patient were €5131 (Q1: 4296; median: 4942; Q3: 5706) in the ED visit group and €5113 (Q1: 4500; median: 4954; Q3: 5484) in the DA group.

The calculated ICER (in the initial sample data set) was €-2788 per ED return visit averted (Table 1). After bootstrap resampling, the ICER was €-4249 (95%CI= -66,001; +45,547) per ED return visit prevented.

**Table 1:** Calculation of the incremental cost-effectiveness ratio (ICER)

	Rate of ED return visits averted		Costs per patient	
**Groups**	DA	ED	DA	ED
**Results**	0,967329154	0,960797965	5112,864	5131,075
**Difference**	0,006531189		-18,211	
**ICER at baseline**	€-2788 per ED return visit averted			

DA: Direct admission group; ED: Emergency department group

**Fig. 1:**
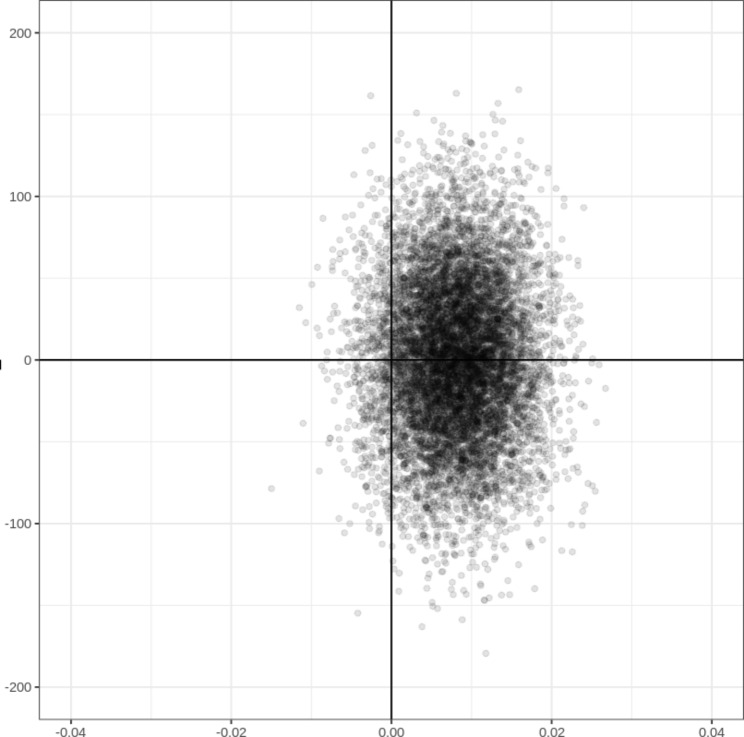
Cost-effectiveness plane (5000 bootstrap replications)

On the cost-effectiveness plane (Figs. 1), 59.8% of the 5000 obtained ICERs were situated in the south–east quadrant (DA was dominant, i.e., more effective and less costly than admission after ED visit), 30.7% in the north-east quadrant (DA was more effective and more costly), 6.6% in the south-west quadrant (DA was less effective and less costly) and 2.9% in the north-west quadrant (DA was less effective and more costly).

**Fig. 2:**
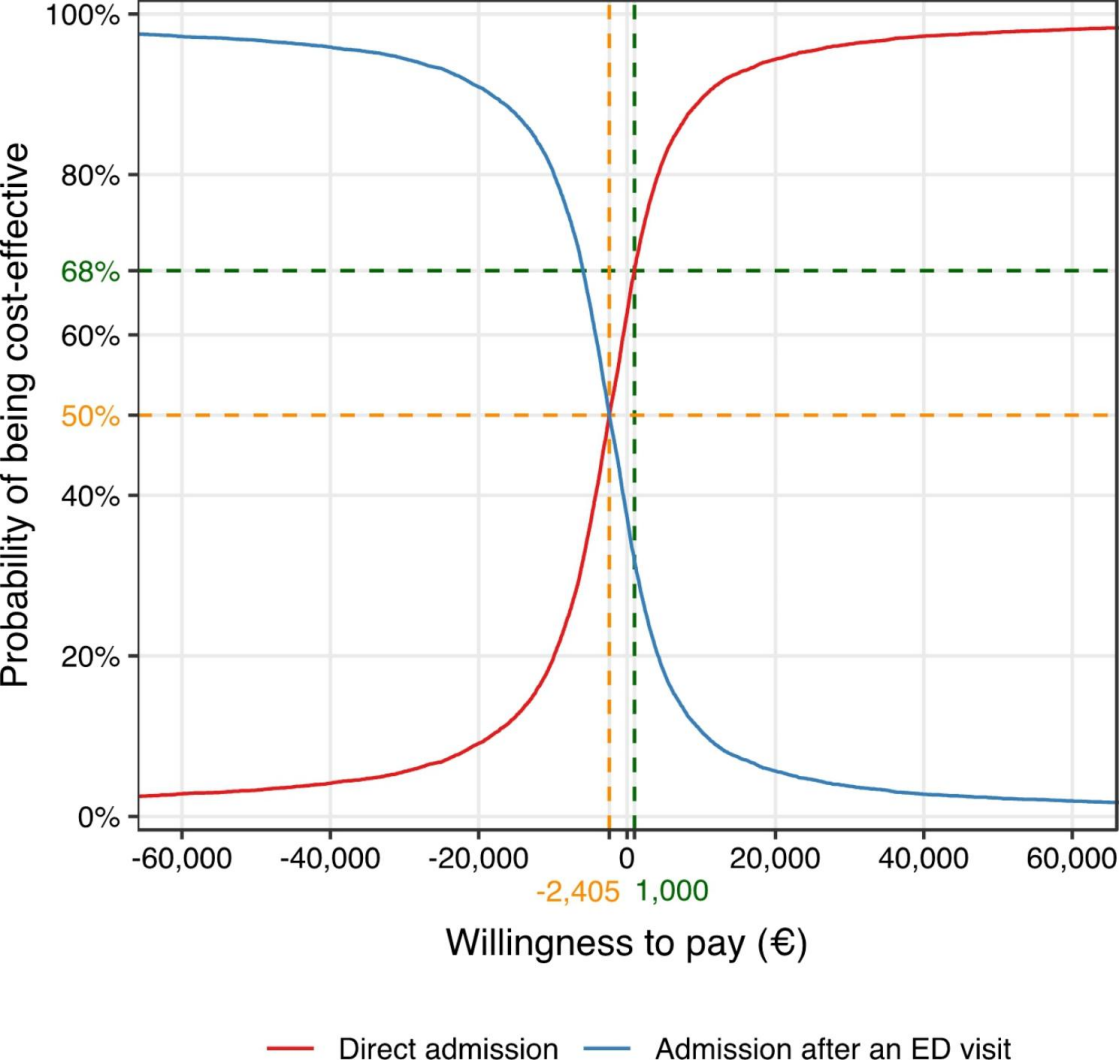
Cost-effectiveness acceptability curves

The CEACs (Fig. 2) show that DA and admission after an ED visit have equal probabilities of being cost-effective at a threshold of €-2405 per ED return visit avoided. Beyond this threshold, DA has a higher probability of being cost-effective. For example, at a threshold of €0 per ED return visit averted, the probability of being cost-effective is 63% and at a threshold of €1000 per ED return visit prevented, the probability of being cost-effective is 68%.

## Discussion

In our previous APHP direct-admission study, DA to an AGU was associated with greater effectiveness (lower hospital LOS, as well as lower likelihood of post-acute care transfer, including follow-up and rehabilitation care) than admission to an AGU after an ED visit [19]. No significant association was found with the risk of ED return visit [19]. In this economic evaluation, we aimed to assess the cost-effectiveness of DA to an AGU versus admission after an ED visit for the elderly to avoid a return ED admission. At baseline, we found a negative ICER (€-2788 per ED return visit averted), which means that DA was more effective in avoiding an ED return visit and less costly than admission after an ED visit. An acceptability curve showed that DA can be considered a cost-effective intervention at a threshold of €-2409 per ED return visit averted. It also demonstrated that if the payer is not willing to pay additional euros per ED return visit avoided, DA is cost-effective in 63% of cases, i.e., 63% of the 5000 ICERs are situated in the south-east quadrant. Thus, our results are strongly in favor of DA implementation. However, it should be remembered that they cannot be applied to all elderly patients presenting to the ED, as the calculations were based on data obtained from a population in which patients with severe acute illness were excluded.

To our knowledge, this study is the first cost-effectiveness analysis of DA to an AGU for elderly patients, compared with admission after an ED visit. Some observational studies have already shown that admissions to AGUs (compared with non-geriatric units) are associated with better outcomes and lower costs [25, 26]. Another study, conducted on nearly 1 million ED visits resulting in over 187 acute care hospitalizations in California, found that periods of ED overcrowding were associated with 1% increased costs per admission [27]. However, none of these studies reported ICERs, which are nonetheless essential to inform stakeholders’ decision-making. In a context of limited resources, decision makers must consider the allocation of resources. If €100 is allocated to a new health program, for example to gain an additional unit of effectiveness due to the implementation of such a program (here, an ED return visit averted thanks to the implementation of DA to an AGU), it implies that the same €100 cannot be allocated to a competing health program (in the same or alternative field of health) [28]. This is considered to be the opportunity cost [23]. Because of our analysis of the uncertainty surrounding the cost-effectiveness ratios, it should be borne in mind that in 37% of cases, the payer will have to be willing to pay additional euros if they choose to favor DA to an AGU over admission after an ED visit. It is difficult to define what is an acceptable incremental cost-effectiveness ratio. The threshold for willingness to pay may vary depending on the context in which decisions are made, and this may be different between countries due to different health policies, organization, and financing of health care. We therefore used analytical tools such as acceptability curves, a guarantee that cost-effectiveness studies were of good quality, which can inform decision makers about the likelihood that a new health program may be cost-effective, based on a variety of the Willingness to Pay schedule. If we extend the reasoning, as Bourel et al. did in a cost-effectiveness analysis in a completely different field of care, should the payer decide to invest €100,000 in the DA of elderly patients to the AGU rather than continuing to hospitalize this cohort via the emergency room, there is a 68% chance of averting 100,000/1000 = 100 ED return visits [28]. The results of such economic calculations favorable to the implementation of DA of elderly people to the AGU are reinforced by the fact that this group of patients is less likely to be discharged in follow-up and rehabilitation care than those admitted after an ED [19]. Indeed, the daily hospitalization cost in follow-up and rehabilitation care is high, and the LOS is often long, on average 35 days in 2019 [29], before the patient returns to the institution or home.

While the results of the economic analysis are important to consider when choosing one intervention over another, there are other important considerations, such as the feasibility of DA intervention, especially in hospitals with problems related to access block and ED overcrowding [19]. Increasing the total number of AGU beds, as well as follow-up and rehabilitation care beds, might be important levers [30–34]. In a large study involving 17,111 patients experiencing acute hospital discharge delays in Canada [32], patients waiting for nursing home admission accounted for 41.5% of such bed days while only accounting for 8.8% of acute hospital discharge delay patients. This means that a small number of patients with non-medical days waiting for nursing home admission contribute to a substantial proportion of total non-medical days in acute hospitals. Some authors described the end of acute hospitalization as “push” rather than “pull” systems, patients being pushed to the next stage by pressure of patients behind them rather than pulled to the next stage [34]. Higher availability of follow-up and rehabilitation care beds might help the transition to a “pull” system. Increasing the number of both AGU and follow-up and rehabilitation care beds would lead to an obvious increase in a hospital’s functioning costs. However, according to the results of our study, these investments could be offset by the costs of ED return visits averted and related re-hospitalizations. Feasibility of DA is also related to better management of patient flow over the entire geriatric pathway. General practitioners should play an important gatekeeping role for DA, but this is conditional on their availability. In Norway, which has a gatekeeper-based healthcare system, Blinkenberg et al. found that only 65% of the emergency-admitted patients came through the primary healthcare gatekeeping system (general practitioners and out-of-hours doctors) [35]. DAs were more common in central areas (45%), where only 18% of referrals were from a GP. Among hospital inpatients admitted for unscheduled care in the UK, patients able to get a general practice appointment on their last attempt were more likely to have been admitted via a GP than after an ED visit [36]. Better coordination between outpatient and inpatient care results in a reduction in avoidable costs [37].

This study has some limitations. The first, already mentioned in the APHP direct-admission study [19], relates to the comparison of effectiveness between the two intervention groups: the choice of DA vs. ED was not randomly assigned, and potential confounding by indication could bias our analyses. IPW weighting based on a propensity score was used to balance baseline characteristics between groups, although unmeasured confounding can never be ruled out in observational studies. Secondly, it could be criticized that when patients had been admitted several times, we analyzed their last admission. As multiple admissions are common in elderly polymorbid patients, this could lead to a loss of data and introduce a selection bias. But on the other hand, taking into account all admissions of these patients with a specific management and prognosis would have overweighted their relative importance and would have had an impact on our overall results with an expected bias towards those of this specific sub-sample. It is why we included previous admissions in the construction of the propensity score. Thirly, we were unable to value hospitalizations in follow-up care and rehabilitation, as we used the APHP Health Data Warehouse, in which patient data were not linked to that regarding follow-up and rehabilitation care in public and private hospitals, most often outside the APHP. The cost implications of this lower hospitalization in the DA group have been discussed above. Finally, whilst we could have considered the societal perspective, the method most often used as it is sufficiently broad to take into account all those affected by the treatments studied, it would have been necessary to estimate travel costs, personal expenses, productivity costs/sick days to qualify for such an analysis [38]. The database we used was not designed for such an analysis and our payer perspective analysis follows Peter J. Neumann’s recommendation, according to which “more attention needs to be paid to the question of what cost data decision makers themselves find most useful” [38].

## Conclusion

The results of this cost-effectiveness analysis of DA to an AGU versus admission after an ED visit for the elderly without severe acute illness argues for directly admitting such patients. Our findings could help support public decision making.

## Data Availability

Data supporting this study can be made available on request (claire.hassen-khodja@aphp.fr), on condition that the research project is accepted by Scientific and Ethical Committee of Assistance Publique – Hopitaux de Paris (AP-HP) clinical data warehouse.

## List of Abbreviations

AGU: Acute geriatric unit
APHP: Greater Paris University Hospitals
CCMA: Classification of Medical Acts
CEAC: Cost-effectiveness acceptability curve
CHEERS: Consolidated Health Economic Evaluation Reporting Standards
CNAM: Caisse Nationale d’Assurance Maladie
DA: Direct admission
ED: Emergency department
GHM: Groupe Homogène de Malades
GHS: Groupe Homogène de Séjours
ICER: Incremental cost-effectiveness ratios
IPTW: Inverse-probability treatment weighting
LOS: Length of stay
PMSI: Program for the Medicalization of Information Systems
